# Strategies to Improve the Thermo-Oxidative Stability of Sunflower Oil by Exploiting the Antioxidant Potential of Blueberries Processing Byproducts

**DOI:** 10.3390/molecules25235688

**Published:** 2020-12-03

**Authors:** Cristina-Ramona Metzner Ungureanu, Mariana-Atena Poiana, Ileana Cocan, Andreea Ioana Lupitu, Ersilia Alexa, Diana Moigradean

**Affiliations:** 1 Faculty of Food Engineering, Banat′s University of Agricultural Sciences and Veterinary Medicine “King Michael I of Romania” from Timisoara, Calea Aradului no. 119, 300645 Timisoara, Romania; cristina_m.ungureanu@yahoo.com (C.-R.M.U.); ileanacocan@usab-tm.ro (I.C.); pag.andreea@yahoo.com (A.I.L.); ersiliaalexa@usab-tm.ro (E.A.); dianamoigradean@usab-tm.ro (D.M.); 2 Faculty of Food Engineering, Tourism and Environmental Protection, “Aurel Vlaicu” University of Arad, Elena Dragoi Street no. 2, 310330 Arad, Romania

**Keywords:** blueberry processing byproducts, freeze-dried extracts, lipid oxidation, high-temperature convective heating, thermo-oxidative stability

## Abstract

This research was conducted in order to establish the effectiveness of two freeze-dried extracts obtained from blueberry processing byproducts resulting from juice manufacturing compared to butylated hydroxytoluene (BHT) in delaying the lipid oxidation of sunflower oil subjected to high-temperature convective heating at 180 °C up to 12 h under simulated frying conditions. The fruits were harvested from spontaneous flora of two regions of Romania, Arieseni (Alba County) and Paltinis (Sibiu County) and the blueberry byproducts extracts (BBE) were noted according to the origin place as ABBE and PBBE. The progress of lipid thermo-oxidation was investigated in terms of peroxide value (PV), *p*-anisidine value (*p*-AV), the response of TBA-malondialdehyde interactions assessed by thiobarbituric acid (TBA) method, the total oxidation (TOTOX) value and inhibition of oil oxidation (IO). The recorded data highlighted that BBE exhibit a high inhibitory response on lipid thermo-oxidation. The inhibitory effect was concentration-dependent, thus, the degree of lipid oxidation was in reverse related to the BBE dose. The exposure of the oil samples supplemented with 800 ppm BBE (ABBE, PBBE) to a high-temperature heating for 12 h led to a significant decrease of the assessed indices compared to additives-free sunflower oil sample as follows: PV (46%; 45%), *p*-AV (21%; 17%), TOTOX (27%; 24%), TBA value (25%; 11%). Regarding the impact of the origin on the potential of BBE to inhibit the lipid oxidative degradation, it was noted that ABBE derived from blueberries grown in a region with a milder climate with moderate precipitations and higher temperatures showed a stronger inhibitory effect on lipid thermo-oxidation than PBBE. A moderate level of 500 ppm BBE inhibited the lipid oxidation similar to 200 ppm BHT. The reported results reveal that BBE represent efficient natural antioxidants that could be successfully applied to improve the thermo-oxidative stability of sunflower oil used in various high-temperature food applications.

## 1. Introduction

Sunflower oil is one of the most often used oils in industrial applications and human nutrition, due to the fact that up to 85% from its composition is represented by unsaturated fatty acids. Due to its unsaturated fatty acids composition, and especially for its content in essential linoleic (9-*cis*, 12-*cis*-octadecadienoic) acid, sunflower oil is considered to be one of the top-quality vegetable oils [1,2]. 

However, sunflower oil is susceptible to thermal degradation that occurs during thermal processing. The high-temperature heating of vegetable oils used in food thermal applications initiates a complex series of chemical reactions eventually resulting in lipid peroxidation [3]. 

The exposure of edible oils to high-temperature heating in various food applications can be achieved with different thermal devices. The convective electric oven is a conventional thermal source used in the thermal food processing by using a fan and exhaust system to blow hot air around the products [4,5]. 

During convective heating, the lipid oxidation is the main degradation process and includes a wide category of complex reactions induced by oxygen in the presence of heat [4]. The result of lipid oxidation during edible oil heat treatment, are mainly related to sensory properties deterioration like undesirable off-odors and off-flavors, a massive loss in nutritive value of the fried product and production of chemical compounds with harmful effect on human health [3,4]. After sunflower oil exposure to thermo-oxidative conditions, during high-temperature heating, following the chemical reactions induced by lipid oxidation, various compounds are sequentially produced [6]. As a result, primary oxidation compounds are produced. From primary oxidation compounds, secondary oxidation compounds will be derived, and so on for tertiary compounds or further oxidation compounds. In result, quality and nutritional value of the both cooking oil as well as fried food products are affected as a result of secondary products formation through the peroxidation of polyunsaturated fatty acids (PUFAs) [3,7]. 

The synthetic antioxidants are widely used to inhibit the oxidative deterioration in vegetable oils but their application has been questioned due to their toxicity and carcinogenicity [8,9]. In the last few years, great efforts are being to improve the oxidative stability of vegetable oils by replacing the synthetic additives with natural antioxidants. Thus, the identifying of new sources of natural bioactive compounds with antioxidant potential represents a great challenge for food industry. Several studies have exposed the potential of vegetable waste as sources of natural bioactive compounds with antioxidant activity due to their high phenolic content [10,11]. 

Berry processing byproducts represent real deposits of valuable substances and can successfully be used as a low-cost extraction material for natural antioxidants. Natural antioxidants can be implemented in food industry as food additives and represent a healthy alternative to synthetic antioxidants [12,13,14]. A high content in phenolic compounds makes berry processing wastes suitable for preventing food and oils lipid oxidation [10,15]. 

The antioxidant activity of the extracts obtained from fruit processing byproducts has been previously proved by different complex methods such as 1,1-diphenyl-2-picrylhydrazyl (DPPH) free radical scavenging assay, ferric reducing antioxidant power (FRAP) assay, cupric reducing antioxidant capacity (CURPAC) assay, Trolox-equivalent antioxidant capacity (TEAC) assay, total radical trapping antioxidant parameter (TRAP) assay, oxygen radical absorbance capacity (ORAC) assay, 2,2′-azinobis-(-3-ethylbenzothiazoline-6-sulfonic acid) (ABTS) radical scavenging assay and lipid peroxidation inhibition capacity (LPIC) assay [16,17,18,19,20]. Depending on the mode of action of the antioxidant compounds, these methods evaluate the capacity of antioxidants to quench the free radicals or the ability of antioxidants to reduce the oxidants [16]. Regarding the total phenolic compounds investigation, Folin-Ciocalteu spectrophotometric assay is the most common method used to quantify the total phenolic content and high-performance liquid chromatography (HPLC) is often used in profiling the individual polyphenolic compounds [16,17,18,19,20].

The effect of berry extracts on lipid oxidation has been investigated mostly on meat products [21,22,23,24]. Ganhão et al. [22], performing research on several Mediterranean berries, demonstrated that the berries extracts were effective in inhibiting lipid oxidation in raw, cooked and cooked and chilled porcine burger patties. Varzaru et al. [23] reported that extracts obtained from bilberry, cranberry and raspberry leaves had an inhibitory effect on lipid peroxidation on chickens’ meat. Burri et al. [24] proved that bilberry and red currant extracts, are efficient in lipid oxidation inhibition in a processed meat model system.

Although the reported results recommend the valorization of fruit processing byproducts as a source of bioactive compounds with antioxidant properties, so far there are limited data concerning the inhibitory effect of various berries processing byproducts against lipid oxidation developed in vegetable oils exposed to thermal stress. To our knowledge, this study represents one of the few attempts to assess the potential of berry byproduct extracts to retard the lipid oxidation of vegetable oils during thermal treatments. 

The valorization of berries processing byproducts opens new trends to exploit the potential of high values phenolic compounds with proved antioxidant properties in order to be further used in various food applications. Also, it might provide a cost-effective strategy to inhibit the lipid oxidation in vegetable oils used in various high-temperature food applications.

This research was planned to assess the efficiency of natural extracts obtained from blueberry processing byproduct resulted to juice manufacturing, to inhibit the lipid oxidation in sunflower oil subjected to high-temperature heating. In this regard, a few important practical questions are addressed: What are the differences regarding the inhibitory effect of freeze-dried extracts compared to butylated hydroxytoluene (BHT) on lipid oxidation process developed in sunflower oil samples expose to high-temperature heating? What are the recommended specific doses of extracts applied in sunflower oil to ensure an inhibitory effect similar to that of BHT? Are there some differences in the antioxidant properties of the freeze-dried extracts related to the blueberries origin area that impact their effectiveness to inhibit the lipid thermo-oxidative degradation?

To answer these questions, the progress of lipid thermo-oxidation developed in sunflower oil samples exposed to high-temperature was monitored by chemical indices, such as peroxide value (PV), *p*-anisidine value (*p*-AV), total oxidation value (TOTOX), and inhibitory effect (IO). In addition, thiobarbituric acid (TBA) assay was applied to evaluate the potential of freeze-dried blueberry byproduct extracts to inhibit the thermo-oxidative degradation process of sunflower oil sample during high-temperature exposure.

## 2. Results and Discussion

This study was performed on additives-free refined sunflower oil supplemented by three concentration levels with ABBE, respectively PBBE (i.e., 200, 500, 800 ppm) and butylated hydroxytoluene (BHT) applied as synthetic antioxidant at a level of 200 ppm. In addition, an oil sample without any antioxidant was prepared as a control. Oil samples were subjected to high-temperature convective heating for 12 h under simulated frying conditions, at a temperature of 180 ± 5 °C. The doses of ABBE and PBBE were selected in agreement with previous investigations that established that the inhibitory effect on lipid oxidation increased with antioxidants concentration [4,25]. In order to highlight the inhibitory effect of BHT and BBE on lipid thermo-oxidative degradation, the results were processed by one-way ANOVA test. Data reported after statistical processing revealed the significance of the changes recorded in monitored indices during the high-temperature exposure of sunflower oil samples in response to supplementation with BBE and BHT, relative to the control sample. 

### 2.1. Evaluation of Antioxidant Properties of Freeze-Dried Blueberry Byproducts Extracts

Data exposed in Table 1 reveal the total phenolic content of freeze-dried BBE and their antioxidant activity evaluated by 1,1-diphenyl-2-picrylhydrazyl (DPPH) radical scavenging activity and the ferric reducing antioxidant power (FRAP). 

The residual moisture content of the freeze-dried blueberry byproducts extracts (ABBE and PBBE) was required in order to express their antioxidant properties relative to the dried substance (d.s) content. A low moisture content for both ABBE (3.29%) as well as PBBE (3.71%) has been registered. The low values of moisture content make microbial and enzymatic degradation impossible, thus ensuring an increased shelf life for natural extracts obtained by freeze-drying [19]. 

The statistical analysis of the obtained results revealed significant difference (*p* < 0.05) in antioxidant properties of ABBE compared to PBBE. The TPC values recorded in BBE samples were 134.61 mg GAE/g d.s for ABBE, respectively 119.67 mg GAE/g d.s for PBBE. 

It can be noticed that TPC is with 12% higher in ABBE compared with PBBE sample. In a similar study conducted by Paes et al. [26], the reported TPC value of freeze-dried blueberry waste was 65.52 mg GAE/g. 

The ferric reducing power indicate the antioxidant activity of BBE and express the ability of antioxidant compounds to donate electrons, thus reducing the oxidized intermediates of the peroxidation process [4]. The results recorded after FRAP assay applied on BBE, were 1481.54 μM Fe^2+^/g d.s for ABBE and 1257.06 μM Fe^2+^/g d.s for PBBE. The ferric reducing power is higher with 18% in ABBE compared to PBBE, thus highlighting a superior antioxidant activity in extract obtained from blueberries grown in a region with a milder climate.

DPPH radical scavenging activity express the hydrogen donating capacity of investigated extracts due to the presence of bioactive substances with antioxidant properties. After antioxidant activity investigation of BBE by DPPH assay, the same conclusion emerges as in the case of ferric reducing antioxidant power assessment, i.e., that the antioxidant potential has been higher in the freeze-dried extract obtained from blueberry processing byproducts from Arieseni, the area with a milder climate represented by moderate precipitation regime and higher temperatures, compared to those from Paltinis, the area characterized by a harsher climate with abundant precipitations and lower temperatures. Thus, a loss of 22% in the antioxidant activity value, assessed by DPPH procedure and expressed as mg GAE/100 g d.s, was recorded in PBBE compared to ABBE. 

It can be pointed out that the highest value of antioxidant activity assessed by DPPH and FRAP method has been revealed in the BBE sample with the highest TPC content, respectively in ABBE.

Apart from their capacity to donate hydrogen or electron, antioxidant role of phenolic compounds is also assigned to their stable radical intermediates, which limit especially fatty acids and oils oxidation from food products. According to the lipid substrate, antioxidant activity may vary widely. Hydrophilic antioxidants are more efficient in lipophilic environment providing an excellent stability against lipid oxidation due to their orientation towards the oil-air interface. In a hydrophilic environment, hydrophilic antioxidants tend to dilute and act weakly against lipid oxidation [4].

**Figure 1:**
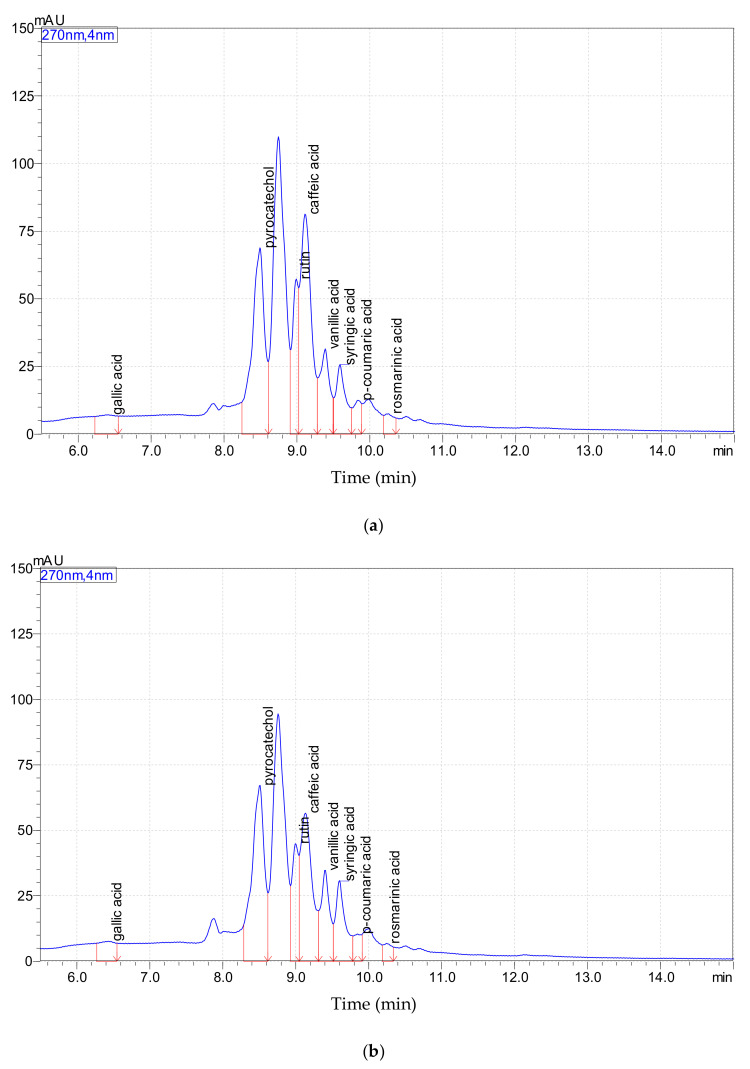
Chromatographic profile of polyphenolic compounds identified in the freeze-dried blueberry byproducts extracts: (**a**) Arieseni blueberry byproducts extract (ABBE); (**b**) Paltinis blueberry byproducts extract (PBBE).

Figure 1 shows the chromatographic profile of the polyphenolic compounds identified in the freeze-dried extracts obtained from byproducts resulting after Arieseni and Paltinis blueberries processing for juice manufacturing. 

The amount recorded for individual polyphenolic compounds by chromatographic analysis is presented in Table 2. 

It can be seen that most of the polyphenolic compounds identified in the freeze-dried extracts obtained from blueberry byproducts were the phenolic acids such as *p*-coumaric acid (*p*-CA), caffeic acid (CA), rosmarinic acid (RA), vanillic acid (VA), gallic acid (GA), and syringic acid (SA). In addition to the phenolic acids, other polyphenolics including rutin (R) and pyrocatechol (PC) have been identified. It can be seen that among them PC has been recorded in the highest amount. 

Our results revealed that the polyphenolic profile of BBE was similar with that of blueberry processing waste reported in the study performed by Lee and Wrolstad [27].

As it may be observed from Table 2 and Figure 1, in the ABBE and PBBE were detected the same polyphenolic compounds, instead, their quantities varied. The statistical analysis of the data exposed significant differences (*p* < 0.05) in polyphenolic compounds quantities identified in the freeze-dried blueberry byproducts extracts. Although most of polyphenolic compounds were identified in a higher amount in ABBE, it was observed that the values recorded for VA and SA were more increased in the PBBE sample. This finding proves that the origin area in terms of environmental conditions, could influence the accumulation pattern of polyphenolic compounds. 

As it has been displayed in other studies, the origin area, along with other factors such as fruit variety, ripeness stage, harvest period, plant nutrition, and storage conditions, influence the development of bioactive substances in blueberries and their fractions [17,19].

### 2.2. Impact of Sunflower Oil Supplementation with Freeze-Dried Blueberry Byproducts Extracts and BHT on Thermo-Oxidative Stability

#### 2.2.1. Peroxide Value (PV)

PV of sunflower oil samples subjected to thermal treatment at 180 °C was evaluated in order to reveal the development degree of primary lipid oxidation in response to high-temperature exposure. Hydroperoxides are odorless and colorless compounds generated by primary lipid oxidation. They represent an unstable category of chemical compounds that can suffer both enzymatic and non-enzymatic degradation and produce a multiple variety of secondary products. Generally, the peroxide index is used to determine the lipid oxidation early stages. The peroxide value increases only with the rate of the hydroperoxides formation. As a result, the lipid oxidation process is exceeded by the rate of their decomposition to secondary oxidation products [4,28]. 

Data presented in Table 3 reveal the changes registered in the PV expressed as milliequivalents (meq) of active oxygen/kg oil registered in sunflower oil samples in response to high-temperature exposure up to 12 h. The recorded changes in PV are related to the oil samples supplementation with both BHT, as synthetic antioxidant, as well as BBE, as natural antioxidants. 

As it is revealed by data shown in Table 3, the heating process induced the sunflower oil oxidation and led to a significant increase in the PV. However, the primary lipid oxidation was significantly reduced by the addition of BHT and BBE. During thermal treatment, significant differences (*p* < 0.05) in PV, among control sample and those supplemented with 200 ppm BHT and various doses of BBE, has been noticed. The inhibitory effect on primary lipid oxidation was related to BBE concentration. Thus, the highest concentration of BBE, showed the most significant inhibitory effect in the early stages of lipid oxidation. Our results are consistent with data reported in other studies regarding the inhibitory effect of BHT and natural extracts on primary lipid oxidation [4,29].

The addition of BHT and BBE to the sunflower oil samples led to a decrease of 30% in PV of oil sample supplemented with 200 ppm BHT, respectively 42% and 43% for samples supplemented with PBBE and ABBE at a level of 800 ppm, compared to the value registered in control sample after 3 h of heat exposure when the maximum peroxide value was recorded. 

The results reported by Poiana [4] in a study on enhancing the oxidative stability of sunflower oil subjected to 4 h of high-temperature convective heating by supplementation with grape seed extracts, revealed a continuous increase in the peroxide value, throughout the heating period. 

Thus, at the end of heat exposure it was reported a decrease of peroxide values with 32%, respectively 39%, reported to the control sample, for sunflower oil supplemented with 200 ppm BHT, respectively grape seed extracts applied at a level of 800 ppm, being in agreement with our findings. 

Also, in the research performed by Khor et al. [30] on the palm olein heated at 180 °C up to 24 h, it has been recorded the highest PV at the end of 4 h, while the samples subjected to thermal treatment from 8 to 24 h led to significant decreases in the peroxide value.

Based on our results, as well as those previously reported [4,30], it can be concluded that PV increases in the first hours of high-temperature exposure and afterwards, by prolonging the thermal treatment, PV can decrease due to the fact that the rate of peroxide formation is exceeded by that of its destruction. Our data revealed that the bioactive compounds with antioxidant properties that are found in ABBE displayed a higher potential to prevent the free radicals generation during the initial stage of oxidation, compared with PBBE, thus proving that the blueberries origin area could impacted on the inhibitory effect of the freeze-dried extracts. The inhibitory response of BBE are close related to their antioxidant properties. During high-temperature treatment, both ABBE and PBBE at a concentration of 500 ppm, showed an inhibitory effect against primary oxidation, similar to the response noted in oil sample supplemented with BHT applied to a level of 200 ppm. 

PV as a relevant parameter regarding oils quality, must not exceeded in refined oils the upper limit of 10 milliequivalents (meq) of active oxygen/kg oil, as it was regulated by Codex Alimentarius [31]. The supplementation of sunflower oil with BHT at a level of 200 ppm and various doses (i.e., 200, 500, 800 ppm) of BBE, places the PV recorded in the sunflower oil samples subjected to high-temperature up to 12 h, in the official limits set and contribute in obtaining a superior quality oil.

#### 2.2.2. The Inhibition of Oil Oxidation (IO)

**Figure 2:**
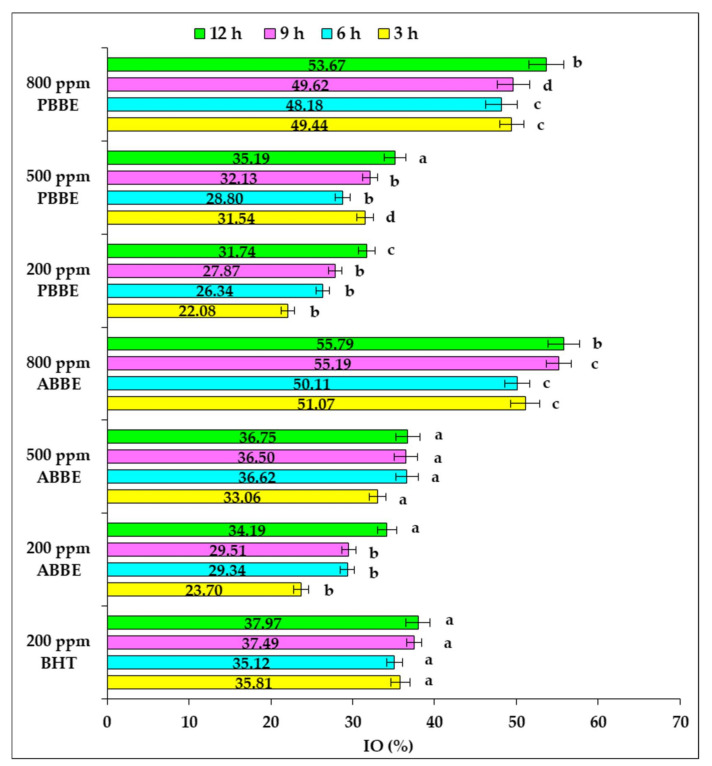
Inhibitory effect of BHT and freeze-dried blueberry byproducts extracts addition on primary lipid oxidation of sunflower oil during high-temperature heating. One-way ANOVA test was used to compare the means differences registered for each heating period among the oil samples supplemented with BHT and different doses of freeze-dried extracts of blueberry byproducts; the values for bars sharing different letters are significantly different (*p* < 0.05); the values for bars sharing the same letters are not significantly different (*p* > 0.05). Results are expressed as the average value of three independent analyses ±SD.

The inhibitory effect (IO) of BBE and BHT on primary lipid oxidation developed in sunflower oil samples in response to high-temperature heating, is displayed in Figure 2. 

Statistical data processing by one-way ANOVA test for any of the investigated heating periods highlighted that the increase in BBE concentration used for sunflower oil supplementation led to a significant increase in IO (*p* < 0.05). 

The reported results revealed that ABBE applied to a level of 500 ppm displayed an inhibitory effect similar to that of 200 ppm BHT. Also, the addition of PBBE in oil sample to a level of 200 ppm inhibited the primary lipid oxidation in a lesser extent than BHT. The addition of ABBE and PBBE in sunflower oil to a level of 800 ppm, showed a higher inhibition of primary lipid oxidation than BHT. Our results revealed that the sunflower oil supplementation with any concentration of BBE have not shown pro-oxidative effect during high-temperature exposure up to 12 h. 

#### 2.2.3. *p*-Anisidine Value (*p*-AV)

Hydroperoxides, as primary products of lipid oxidation process, decomposed further and leads to raising of the secondary oxidation products amounts that are more stable than primary oxidation products during thermal treatment applied to the oil samples [4]. The secondary oxidation products such as aliphatic aldehydes and ketones are responsible for off-odors and off-flavors, i.e., rancid odor and taste, of edible oils. The simultaneous identification of the primary and secondary products generated in response to high-temperature exposure is required for an optimal observation of lipid oxidation. *p*-AV is a valid measurement for secondary oxidation products evaluation [4,32]. 

The changes reported in the *p*-AV of sunflower oil samples supplemented with BHT and various doses of BBE during high-temperature heating are presented in Table 4. It can be noticed that the convective heating at 180 °C promoted the rapid transformation of primary oxidation products in the secondary products that are related to off-odors and off-flavors occurred in the sunflower oil. 

Considering the obtained results, it can be noticed that *p*-AV has been in a continuous increase throughout the high-temperature exposure of the sunflower oil samples. A higher *p*-anisidine value decreases the smoke point that results in obtaining of a poor-quality oil. Supplementation of the sunflower oil subjected to high-temperature heating with BHT and various concentrations of BBE resulted in significant decreases in *p*-AV (*p* < 0.05) compared to the control sample. 

As in the case of the results registered for peroxide value, it could be observed that, both ABBE and PBBE applied at a level of 800 ppm provided the best protection against secondary oxidation of sunflower oil subjected to thermal treatment. These results are consistent with data reported in similar studies of enhancing the oxidative stability of sunflower oil by supplementation with extracts from fruits byproducts [4,33]. In this regard, data reported by El-Hadary and Taha [33] in a study on enhancing the lipid oxidative stability of different edible oils by addition of various levels of pomegranate peel methanolic extracts proved that the increasing of the natural extract concentration resulted in a significant increase in the inhibitory response.

After 12 h of high-temperature exposure, the *p*-AV registered in sunflower oil sample supplemented with 200 ppm BHT was 58.85, resulting in a decrease of about 16% related to the control sample. Depending on the applied freeze-dried extract doses and the blueberries origin area, the addition of ABBE and PBBE to the sunflower oil samples subjected to thermal treatment led to decreases in *p*-AV in the range 6–21% compared with the control sample. The obtained results highlight that the inhibitory response on secondary lipid oxidation was dose-dependent, being close related to the level of polyphenolic compounds from BBE.

Statistical data analysis revealed that the oil supplementation with ABBE, respectively PBBE at a level of 800 ppm PBBE, induced a significantly inhibition (*p* < 0.05) of secondary lipid oxidation. BBE applied at a level of 500 ppm, provided a similar protection against secondary lipid oxidation as BHT applied at a level of 200 ppm. 

It can be observed that at a level of 200 ppm both BBE, induced an inhibitory effect of secondary lipid oxidation lower than BHT at a level of 200 ppm. Contrary, by applying of ABBE and PBBE at a level of 800 ppm, BBE it was obtained a higher inhibitory response compared with that of BHT.

#### 2.2.4. Total Oxidation (TOTOX) Value 

The TOTOX value represents a mathematical prediction for oxidative stability of an oil or fat sample on the base of both indicators of primary and secondary lipid oxidation, PV, respectively *p*-AV. 

**Figure 3:**
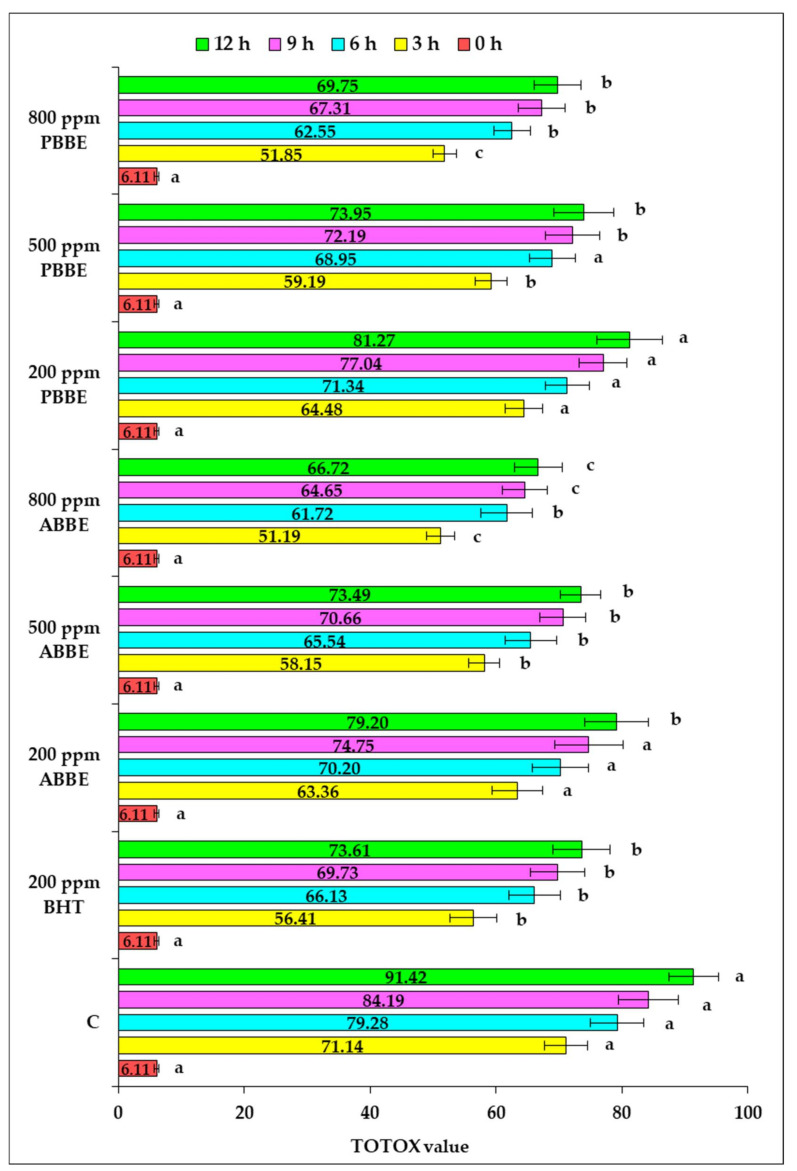
Effect of sunflower oil supplementation with BHT and freeze-dried blueberry byproducts extracts on TOTOX value during high-temperature heating. One-way ANOVA test was used to compare the means differences registered for each heating period among the oil samples supplemented with BHT and different doses of freeze-dried extracts of blueberry byproducts, relative to the control sample; the values for bars sharing different letters are significantly different (*p* < 0.05); the values for bars sharing the same letters are not significantly different (*p* > 0.05). Results are expressed as the average value of three independent analyses ±SD.

In Figure 3 were depicted the changes in the total oxidation value of the sunflower oil samples supplemented with BHT and different doses of BBE, subjected to convective heating at 180 °C up to 12 h.

It can be appreciated that the TOTOX value represents an appropriate index for monitoring the lipid oxidation process developed in edible oils in response to thermal stress. This is a reliable indicator of overall oxidative stability, with its values being successfully correlated with the degree of lipid oxidation [4]. Thus, an increase registered in the TOTOX value indicates a higher level of lipid oxidative deterioration [32]. As it is revealed by data depicted in Figure 3, the TOTOX value increased with extension of high-temperature heating period. It was noticed that the sunflower oil samples supplemented with BHT and various doses of BBE showed significant lower TOTOX values compared with the value recorded in the control sample (*p* < 0.05). After 12 h of convective heating, TOTOX value registered in oil samples supplemented with different concentrations of ABBE, respectively PBBE decreased in the range 11–27% related to the control sample. The highest level of BBE (800 ppm) was the most effective to inhibit the oxidative degradation process of sunflower oil during the high-temperature heating. Also, ABBE applied at a level of 500 ppm, displayed a similar inhibitory effect as the oil sample supplemented with 200 ppm BHT.

So far, only several studies have been investigated the improvement of oxidative stability of sunflower oil by supplementation with natural extracts obtained from berries byproducts or various value-added products obtained from fruits processing wastes. However, the data obtained after TOTOX value calculation are in agreement with the results reported by other studies in which sunflower oil was supplemented with other types of extracts [4,34]. Neves et al. [34] reported a TOTOX value of 69.30 after 27 h of heat exposure at 180 °C of the sunflower oil sample supplemented with 1000 ppm of ethanolic extract obtained from prickled broom. This finding is in agreement with our data, when TOTOX values of 69.75, respectively 66.72 were recorded in response to 12 h of heating at 180 °C of the sunflower oil sample supplemented with 800 ppm PBBE, respectively ABBE. 

Our results revealed that in the sunflower oil system, BBE could limit the lipid thermo-oxidation induced by heat-temperature heating. It was previously reported that the supplementation of sunflower oil with natural extracts contributes to the development of an oil system surrounded by natural antioxidants that were efficient in preventing the thermo-oxidative degradation process due to the location of phenolic compounds on the interface of the lipid system [4]. 

Based on the obtained results it has been found that the improvement of sunflower oil thermo-oxidative stability by supplementation with BBE was concentration-dependent. 

#### 2.2.5. Thiobarbituric Acid (TBA) Value

The TBA assay is a widespread procedure used to evaluate the lipid oxidation process developed in foods, fats and oils both during thermal processing as well as their storage. The TBA method is based on the measurement of malondialdehyde (MDA) amount in foods, the results being close related to the development of unpleasant odors and flavors. Due to the fact that malondialdehyde is responsible for the typical TBA reaction, this test is suitable for assessing the degree of food oxidative rancidity [35,36]. 

In the free form, malondialdehyde is not present in large quantities in the oxidized lipids, but it is released when the system is subjected to thermal treatments at high temperatures. The color intensity in the spectral range 532–540 nm is a measure of the malondialdehyde amount that reflects the degree of lipid oxidation. It has been highlighted that the oils and fats rich in polyunsaturated fatty acids (PUFAs) that contain three or more double bonds, give a strong TBA reaction in the early stages of oxidative rancidity. This reaction is partly due to the secondary oxidation of the primary carbonyl compounds [35]. 

**Figure 4:**
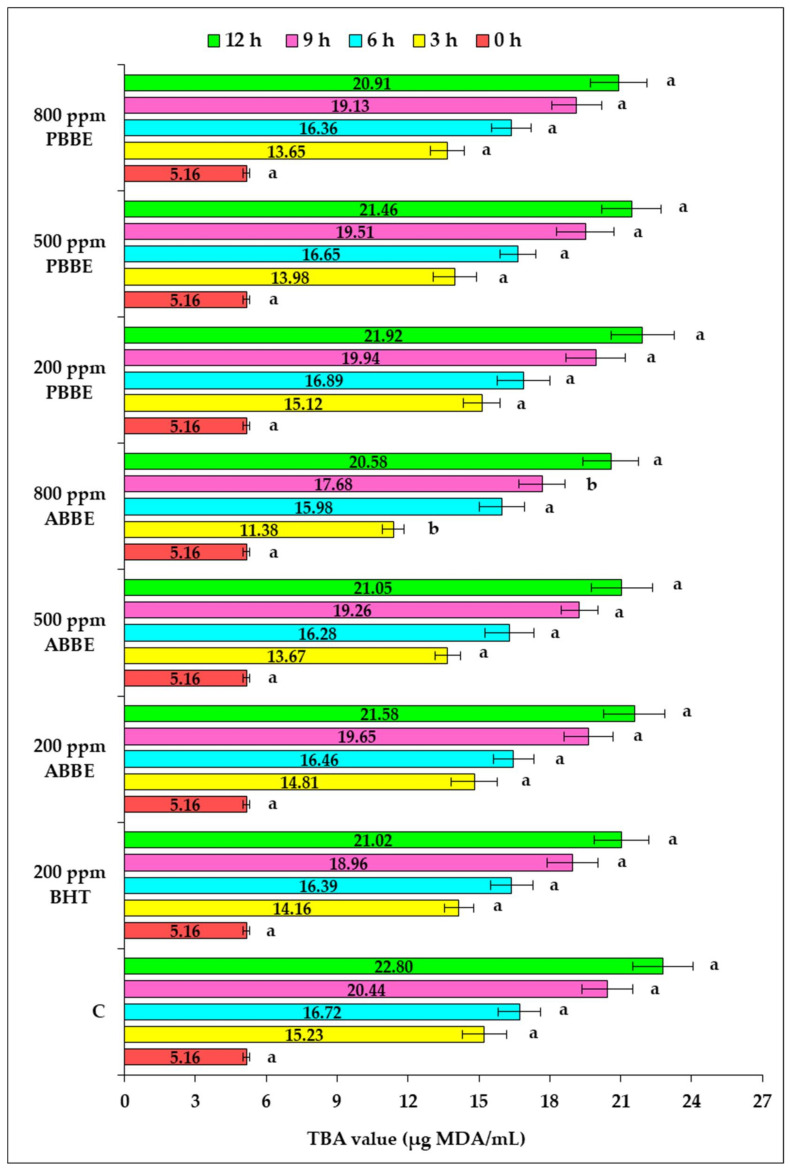
The effect of sunflower oil supplementation with BHT and freeze-dried blueberry byproducts extracts on TBA value during high-temperature heating. One-way ANOVA test was used to compare the means differences registered for each heating period among the oil samples supplemented with BHT and different doses of freeze-dried extracts of blueberry byproducts, relative to the control sample; the values for bars sharing different letters are significantly different (*p* < 0.05); the values for bars sharing the same letters are not significantly different (*p* > 0.05). Results are expressed as the average value of three independent analyses ±SD.

The changes registered in TBA value, expressed as µg MDA/mL oil, in response to high-temperature heating for 3, 6, 9 and 12 h of sunflower oil samples supplemented with BHT and different levels of ABBE and PBBE were shown in Figure 4. 

As an indicator of lipid oxidation, TBA shows the secondary stage of oxidation and the presence of the secondary oxidation products in the investigated sample. A high TBA value indicates an increased oil oxidation and less stability. On the same note, the lower the amount of TBA-malondialdehyde complex is, the lower the absorbance will be, and therefore it means a better protection of the added extracts against secondary lipid oxidation developed in sunflower oil in response to high-temperature treatment.

The results obtained showed that the TBA value was low after 3 h of heat exposure in the all investigated samples due to the fact that the rate of hydroperoxides decomposition is exceeded by the rate of their formation. 

Further, after 6 h of heat exposure, when the hydroperoxides formation has been overcome by their decomposition to secondary oxidation products, the TBA value has increased following the same increasing trend throughout the investigated heating period. Our results pointed out the highest PV at 3 h of heat exposure when the lowest TBA value was recorded, and the lowest PV at the end of thermal exposure when the highest TBA value was recorded. Thus, a direct relationship between the consumption of hydroperoxides and TBA value increasing has been noticed. This finding has been previously reported in other studies on this topic, where a reverse relation between the peroxide value and malondialdehyde content has been pointed out [35,37]. From this point of view, our data are in agreement with the results reported in the study carried out by Okhli et al. [37] on sunflower oil stabilization by addition of citron peels extract, revealing that the hydroperoxides decomposed and produced aldehydes and ketones that led to the increase in TBA value and a decrease in the PV value. Data statistical analysis showed no significant differences (*p* > 0.05) between the investigated samples, excepting for the oil samples enhanced with 800 ppm ABBE where a significant difference was noted after 3, respectively 9 h of high-temperature exposure. After 12 h of heat exposure, the highest ability to prevent the formation of secondary oxidation products was recorded in the sunflower oil samples supplemented with the highest dose of BBE (800 ppm). 

Concerning the impact of the origin place of blueberries on the inhibitory potential of freeze-dried extracts on thermo-oxidative degradation process developed in sunflower oil exposed to high-temperature, it can be noticed that the higher inhibitory effect displayed by ABBE than PBBE applied to any of the investigated doses, throughout the sunflower oil heating, was related to the phenolic compounds content and antioxidant activity of this extract. Our results have proven that, after 12 h of thermal treatment, the most efficient extract in preventing the secondary lipid oxidation was ABBE applied at a level of 800 ppm that induced a decrease in TBA value by 10% compared to the control sample.

## 3. Materials and Methods 

### 3.1. Processing of Blueberry Byproducts Extracts 

Blueberries (*Vaccinium myrtillus* L.) were collected in the year 2018, from two different Romanian areas: Arieseni, Alba County (46°28′35” North, 22°45′25” East) and Paltinis, Sibiu County (45°39′10” North, 23°55′55” East). Arieseni area is characterized by a milder climate with moderate precipitation regime and higher temperatures, while the Paltinis area is represented by a harsher climate with abundant precipitations and low temperatures. The harvest period was the end of June when the blueberries were at maturity stage (fully ripe fruit). Blueberries were afterwards selected and separated of impurities and stored in sealed plastic containers at −20 °C until further use. 

The conditioning of byproducts obtained by juice centrifugal extraction from frozen blueberries, in laboratory conditions using a juice extractor (MES3500, 700 W, Bosch GmbH, Stuttgart, Germany) following a simple procedure, usually applied at household level, were performed according to Metzner Ungureanu et al. [19]. Thus, the blueberry byproducts were conditioned by a convective drying at 60 °C for 12 h in an electric oven (Esmach SpA-Ali Group/Italy, 1200 W, 50 Hz) then grinded using a laboratory mill (Grindomix Retsch GM200, Haan, Germany). 

In order to extract bioactive compounds from blueberry byproducts, a maceration solvent extraction was used. The solid:solvent extraction ratio was 1:10 (*w*/*v*) and the extraction solvent was a hydro-alcoholic ethanol/water mixture (1:1, *v*/*v*) [19]. 

The extraction procedure was performed at 20 °C for 48 h. The mixture obtained was percolated through a 0.45-µm PTFE membrane filter and a clear ethanolic extract was obtained. The clear extracts were evaporated under reduced pressure at a temperature of 50 °C until 100 mL, using a rotary evaporator (RV 10 auto pro V, IKA England Ltd., 100 W, 50 Hz). Further, the blueberry byproducts extracts were subjected to freeze drying using a lyophilizer (Alpha 1-2 LDplus, 230 V/50 Hz, MARTIN CHRIST Gefriertrocknungsanlagen GmbH, Osterode, Germany). After freeze drying, the extracts were maintained at a frozen temperature of −18 °C until other analyses were applied. According to the origin place of blueberries, the freeze-dried extracts obtained from juice processing byproducts were expressed as ABBE for Arieseni area, respectively PBBE for Paltinis region.

### 3.2. Application of Freeze-Dried Blueberry Byproducts Extracts to Sunflower Oil

Sunflower oil, freshly refined and free of synthetic antioxidants, was divided into eight portions. Six of them, were supplemented with 200, 500 and 800 ppm freeze-dried blueberry byproducts extracts (ABBE and PBBE), the seventh portion was supplemented with butylated hydroxytoluene (BHT) applied at a legal limit of 200 ppm, and the eighth portion of sunflower oil, without any additive, was used as a control sample (C). 

To ensure the diffusion of extracts and BHT in the sunflower oil, before applying, the supplements were diluted in a minimum volume of absolute ethanol in an ultrasonic water bath and then were mixed with the oil for 10 min, followed then by a vacuum evaporation. The control sample was prepared in the same conditions [4]. 

### 3.3. High-Temperature Heating of Sunflower Oil

To assess the inhibitory effect of BBE and BHT on thermo-oxidative lipid degradation, the sunflower oil samples were subjected to heating for 3, 6, 9 and 12 h at a temperature of 180 °C in simulated frying conditions, using a convection electric oven (Esmach SpA-Ali Group, 1200 W, 50 Hz, Grisignano di Zocco, Italy). In this purpose, 25.0 ± 0.5 g oil samples were weighed in Pyrex Petri dishes with 9 cm inner diameter and placed in the electrical convection oven regulated at 180 ± 5 °C. Separate oil samples were used for different heating period. The oil samples obtained at the end of each thermal treatment period were rapidly cooled and stored until further analysis at a freezing temperature of −18 °C. Also, after each heating period, the oven was stopped for 1 h with the door widely open, to be completely cooled down before starting another heating period.

### 3.4. Freeze-Dried Blueberry Byproducts Extracts Analysis

#### 3.4.1. Moisture Content 

The moisture content of the freeze-dried extracts obtained from blueberry processing byproducts (ABBE and PBBE) was determined according to the method 925.09 of the AOAC [38].

#### 3.4.2. Total Phenolic Content 

Total phenolic content (TPC) of BBE was spectrophotometrically determined using the Folin-Ciocalteu method, as it was previously reported in the literature [19,39,40]. The samples containing polyphenolic compounds, prepared from the freeze-dried extracts, are reduced by the Folin-Ciocalteu reagent by generating a blue colored complex. The depth of the produced blue color is closely related to the content of polyphenolic compounds. Thus, a deeper color results in a higher absorbance and consequently, a higher polyphenolic compounds content [40,41]. For this purpose, 40 mg of freeze-dried extracts were dissolved in 50 mL ethanol/water mixture (1:1, *v*/*v*). An aliquot of 1 mL of each obtained mixture, previously diluted in a ratio of 1:25 with distilled water, was further used in analysis. Gallic acid in concentrations ranging from 20 to 200 mg/L, was used as a standard to prepared the calibration curve [19]. The absorbance was measured using a double-beam UV-Vis spectrophotometer (Specord 200, Analytik Jena Inc., Jena, Germany) at 765 nm, against a blank sample prepared in the same conditions. TPC was expressed as mg gallic acid equivalent (GAE)/g dried substance (d.s). All determinations were performed in triplicate and the results were reported as mean value ± standard deviation (SD).

#### 3.4.3. Antioxidant Activity 

The antioxidant activity of the freeze-dried extracts has been investigated by a 1,1-diphenyl-2-picrylhydrazyl (DPPH) radical scavenging assay as well as by ferric reducing antioxidant power (FRAP) assay.

##### DPPH Assay

Free radical scavenging ability of ABBE and PBBE was tested by DPPH assay using the stable radical 1,1-diphenyl-2-picrylhydrazyl (DPPH) according to the previously described method [19,42,43]. 

This assay is based on the spectrophotometric measurement at a wavelength of 517 nm of the changes recorded in the DPPH radical concentration occurring as a result of DPPH radical reaction with an antioxidant compound. Positive controls with concentrations from 2.5 to 50 mg/L gallic acid prepared in 96% ethanol were used as a reference. The DPPH radical scavenging activity of the freeze-dried extracts was expressed as mg GAE/100 g d.s. Also, the percentage of DPPH inhibition, I (%), was determined in agreement with Metzner Ungureanu et al. [19]. All determinations were performed in triplicate and the results were reported as mean value ± SD. 

##### FRAP Assay

The ferric reducing ability of ABBE and PBBE was investigated by FRAP assay according to the method described by Benzie and Strain [44]. Thus, 0.1 g of BBE were treated with 20 mL ethyl alcohol 70% (*v*/*v*) for 10 min, then, the solution was filtered and the obtained filtrate was used for analysis. The FRAP assay is based on the ability of antioxidant compounds to reduce the ferric ion (Fe^3+^) to ferrous ion (Fe^2+^) in the presence of tripyridyltriazine (TPTZ) by forming an intense blue Fe^2+^—TPTZ complex with a maximum absorbance at 593 nm [44]. 

The reduction process has been monitored by the changes in the absorbance value at 593 nm that were linear over a concentration range from 0.1 to 1.0 µM Fe^2+^ equivalents/mL. A UV-Vis double-beam spectrophotometer Specord 200 from Analytik Jena Inc. (Jena, Germany) was used to measure the absorbance at the mentioned wavelength. 

The FRAP value of the freeze-dried extracts was expressed as µM Fe^2+^ equivalents per g d.s. All determinations were performed in triplicate and the results were reported as mean value ± SD.

#### 3.4.4. Polyphenolic Compounds Profile Evaluation of Freeze-Dried Blueberry Byproducts Extracts by Chromatographic Analysis 

The polyphenolic compounds profile of the freeze-dried extracts was investigated by chromatographic analysis using an ultra-high-performance liquid chromatograph (UHPLC), Shimadzu Nexera X2 (Tokyo, Japan), equipped with a diode array detector M30A Shimadzu (Tokyo, Japan) and a reverse phase column Nucleosil 100-3-C18 (4 mm inner diameter × 125 mm column length, 3 µm particle size, Macherey-Nagel GmbH, Düren, Germany), according to the previously described method [19,45]. The elution solvents consisted of 0.1% aqueous solution of trifluoroacetic acid with pH = 3 (A), respectively acetonitrile (B). The gradient elution program was established in agreement with the method reported by Metzner Ungureanu et al. [19] and Lupitu et al. [45]. The polyphenolic compounds detected in the spectral range of 200–600 nm were expressed as mg/100 g d.s. All determinations were performed in triplicate and the results were reported as mean value ± SD. 

### 3.5. Evaluation of Lipid Oxidation

The progress of thermo-oxidative degradation of sunflower oil was monitored by determining the commonly used chemical indices such as peroxide value and *p*-anisidine value. In addition, total oxidation and inhibition of the oil oxidation were calculated and TBA method was applied to estimate the antioxidant effect of the freeze-dried extracts against thermo-oxidative degradation processes that occur in response to sunflower oil heating at a high-temperature. 

#### 3.5.1. The Peroxide Value (PV) 

The procedure applied for PV determination is a basic method used in the quality control of the deep frying oils. The peroxide index represents the peroxide content in oils or food products, which through oxidation, release the iodine from a potassium iodide (KI) solution [46,47]. Thus, the peroxide value has been evaluated using the iodometric titration according to the standard methods for oils analysis and it provides information about the occurrence of primary lipid oxidation [48]. Briefly, to 5 g of oil sample was added 25 mL chloroform/glacial acetic acid (2:3, *v*/*v*) mixture. Then, the mixture was shaken in order to dissolve the oil, and 1 mL of saturated KI solution was added. After 1 min of incubation in the dark followed by 5 min of rest, the mixture was diluted with 75 mL distilled water and the generated iodine was titrated with 0.01 N sodium thiosulfate (Na_2_S_2_O_3_) solution, in the presence of starch (10%) that acts as an indicator. The control sample, free of oil, was prepared in the same conditions [48]. 

The PV was calculated according to the relation displayed in the Equation (1):

(1) $$PV(mEqO_{2}/\mathrm{kg}\;\mathrm{oil})=\frac{(V_{1}-V_{2})\times N\times 1000}{W}$$

where V_1_ represents the titration volume of sample (mL), V_2_ represents the titration volume of the control sample (mL), N is the normality of the Na_2_S_2_O_3_ solution and W represents the sample weight (g).

The results of peroxide value were expressed as milliequivalents of active oxygen per 1000 g oil sample (mEq O_2_/kg oil).

#### 3.5.2. The Inhibition of Oil Oxidation (IO)

The inhibition of the oil oxidation (IO) is an indicator for primary oxidation. Calculation of the IO is based on the values recorded in PV determination. 

The percentage of the oil oxidation inhibition was determined according to the relation displayed in the Equation (2) [4]:

(2) $$IO(\%)=(1-\frac{increase\;in\;PV\;of\;oil\;sample}{increase\;in\;PV\;of\;control})\times 100$$

where, by increase in PV of oil sample is meant the increase recorded in the peroxide value of oil samples subjected to different periods of heating reported to the PV recorded at the time 0 in the same sample, while the increase in PV of control represents the increase registered for control sample subjected to different periods of heating, reported to the PV recorded for the control sample at time 0. 

#### 3.5.3. The *p*-Anisidine Value (*p*-AV) 

The *p*-anisidine value has been spectrophotometrically determined according to the standard methods for oils analysis and it represents a reliable indicator to assess the occurrence of secondary products of lipid oxidation [48]. *p*-AV represent a measure of the carbonyl content in oils and is based on the reactivity of the aldehyde carbonyl bond on the *p*-anisidine amine group that leads to the production of a Schiff base with a maximum absorbance at 350 nm wavelength [49,50]. For measuring the *p*-AV, 2 g of oil sample was dissolved in 25 mL isooctane. The absorbance of this solution was measured against a blank isooctane sample at 350 nm using a double-beam UV-Vis spectrophotometer (Specord 200, Analytik Jena Inc., Jena, Germany). An aliquot of 5 mL of obtained solution, respectively 5 mL of isooctane solvent used as a blank, were separately transferred in two test tubes and, then, 1 mL of *p*-anisidine/glacial acetic acid (0.25%, *w*/*v*) solution was added. After 10 min, the absorbance of the oil solution from the first test tube was measured at 350 nm against the solution from the second test tube containing solvent with *p*-anisidine. The *p*-anisidine value was calculated according to the relation displayed in the Equation (3):

(3) $$p\text{-}AV=25\times \frac{1.2\times A_{2}-A_{1}}{W}$$

where A_1_ represent the absorbance of 2 g oil sample in 25 mL isooctane measured against a blank of isooctane, A_2_ represent the absorbance of 2 g oil sample in 25 mL isooctane with 1 mL *p*-anisidine solution measured against a sample of isooctane containing *p*-anisidine solution and W represents the sunflower oil sample weight (g).

#### 3.5.4. The Total Oxidation (TOTOX) Value 

The total oxidation (TOTOX) value was evaluated in order to estimate the oxidative degradation of lipids. 

The TOTOX value reflects the contribution of both peroxide value (PV) and *p*-anisidine (*p*-AV) value to the total lipid oxidation and was calculated according to the following Formula (4) [4,51]:

(4) TOTOX=2×PV+p-AV

#### 3.5.5. Thiobarbituric Acid (TBA) Assay 

Thiobarbituric acid (TBA) assay was used to evaluate the potential of freeze-dried blueberry byproducts extracts to delay thermo-oxidative degradation of sunflower oil during heating process. The TBA method is based on the reaction of malondialdehyde (MDA) with TBA and is applied specifically in the evaluation of lipids secondary oxidation [52,53]. 

TBA method was assessed spectrophotometically according to the slightly modified procedure of Singh et al. [54]. Thus, 2 g oil samples were mixed with 5 mL benzene and 4 mL aqueous TBA solution (0.67%, *w*/*v*). The mixtures were shaken for 30 min using a mechanical orbital stirrer. Next, the samples were left to repose for 10 min to separate the phases. Further, the supernatant was collected in test tubes and heated at 80 °C for 45 min using a water-bath. Then, after the samples have reached the room temperature, the absorbance of the supernatant was measured at 540 nm using the Specord 200, Analytik Jena Inc. (Jena, Germany) spectrophotometer. Spectrophotometric measurements were performed against an oil-free control sample. The calibration curve was achieved by measuring the absorbance of MDA solutions with different concentrations ranging from 10 to 50 µg/mL. The MDA amount in the samples was calculated based on the calibration curve, depending on the absorbance read and the sample weight. The TBA value was expressed as µg MDA/mL oil sample.

### 3.6. Statistical Data Analysis

All analyzes were carried out in triplicates and the results were expressed as mean values ± standard deviation (SD). Analysis of variance (ANOVA one-way) for statistical data analysis, was used. Computations Tukey post-hoc means comparisons and Levene′s test for equal variance were included to estimate the statistical significance of variations among means. Data within the same row or column in the case of tables, or bars, when referring to charts, sharing different superscripts or letters are significantly different (*p* < 0.05). Data within the same row or bars, sharing the same superscripts or letters, are not significantly different (*p* > 0.05). JASP computer software (JASP Team, University of Amsterdam, The Netherlands, version 0.11.1, 2019) was used to perform statistical data processing.

## 4. Conclusions

Data reported in this study revealed that freeze-dried blueberry byproduct extracts showed a high inhibitory effect against primary and secondary lipid oxidation of sunflower oil subjected to convective heating for 12 h at 180 °C. Supplementation with different doses of BBE and 200 ppm BHT resulted in an increased stability of sunflower oil exposed to a high temperature specific to some applications in the food industry. The effectiveness of BBE to improve the thermo-oxidative stability of sunflower oil during heat exposure was close related to the extracts dose. Samples of sunflower oil supplemented with BBE to a level of 500 ppm exhibit an inhibitory response similar with that of BHT, while a level of 200 ppm BBE prevents the thermo-oxidative degradation of sunflower oil less than 200 ppm of BHT. Both ABBE and PBBE applied to a level of 800 ppm, showed a higher inhibitory response than BHT applied at a concentration of 200 ppm. A direct relationship has been noticed between the consumption of hydroperoxides, as primary lipid oxidation products, in order to produced aldehydes and ketones as secondary oxidation products, and the increase of TBA value. Regarding the impact of the fruit origin area on the effectiveness of BBE in limiting the oxidative degradation process in sunflower oil samples subjected to high-temperature heating, it has been recorded a higher inhibitory effect in oil samples supplemented with extract coming from blueberries grown in a region characterized by a milder climate with moderate precipitation regime and higher temperatures. Antioxidant activity of BBE against thermal-oxidation of sunflower oil was related to the presence of polyphenolic compounds. These results prove that BBE, due to their high content in bioactive compounds, are recommended as valuable natural antioxidants for enhancing the thermo-oxidative stability of sunflower oil used in high-temperature food thermal applications. These results may be useful for the edible oil industry in order to replace synthetic preservatives with natural antioxidants to prevent the oxidative lipid degradation in response to thermal exposure.

## Figures and Tables

**Table 1:** Antioxidant characteristics of freeze-dried blueberry byproducts extracts.

Sample	TPC (mg GAE/g d.s)	FRAP (µM Fe^2+^/g d.s)	DPPH	
			I (%)	(mg GAE/100 g d.s)
ABBE	134.61 ± 0.65 ^a^	1481.54 ± 7.42 ^a^	94.75 ± 0.57 ^a^	1789.67 ± 8.43 ^a^
PBBE	119.67 ± 0.71 ^b^	1257.06 ± 6.87 ^b^	92.86 ± 0.49 ^b^	1387.86 ± 7.96 ^b^

One-way ANOVA test was used to compare the means differences registered among samples; data within the same column sharing different superscripts are significantly different (*p* < 0.05); data within the same column sharing the same superscripts are not significantly different (*p* > 0.05). Results are expressed as the average value of three independent analyses ±SD.

**Table 2:** Polyphenolic compounds profile of freeze-dried blueberry byproducts extracts.

Polyphenolic Compounds	Compound Content (mg/100 g d.s)	
	ABBE	PBBE
R	676.75 ± 3.59 ^a^	572.06 ± 3.72 ^b^
PC	13962.14 ± 17.75 ^a^	12423.60 ± 18.04 ^b^
*p*-CA	1682.35 ± 8.72 ^a^	1485.67 ± 7.28 ^b^
CA	1936.13 ± 9.78 ^a^	1660.15 ± 7.47 ^b^
RA	1404.25 ± 7.44 ^a^	1363.01 ± 9.41 ^b^
VA	1272.07 ± 8.39 ^b^	1402.14 ± 8.24 ^a^
GA	205.14 ± 1.42 ^a^	170.24 ± 1.26 ^b^
SA	722.22 ± 5.13 ^b^	861.80 ± 6.29 ^a^

Rutin (R); pyrocatechol (PC); *p*-coumaric acid (*p*-CA); caffeic acid (CA); rosmarinic acid (RA); vanillic acid (VA); gallic acid (GA); syringic acid (SA). One-way ANOVA test was used to compare the means differences registered among samples; data within the same row sharing different superscripts are significantly different (*p* < 0.05); data within the same row sharing the same superscripts are not significantly different (*p* > 0.05). Results are expressed as the average value of three independent analyses ±SD.

**Table 3:** The impact of sunflower oil supplementation with BHT and freeze-dried blueberry byproducts extracts on the peroxide value (PV) during high-temperature heating.

Sample	PV (meq O_2_/kg Oil)				
	Heating Time (h)				
	0	3	6	9	12
C	1.81 ± 0.05 ^a^	11.64 ± 0.47 ^a^	11.15 ± 0.43 ^a^	10.96 ± 0.41 ^a^	10.79 ± 0.38 ^a^
200 ppm BHT	1.81 ± 0.05 ^a^	8.12 ± 0.26 ^d^	7.87 ± 0.23 ^b^	7.53 ± 0.25 ^b^	7.38 ± 0.31 ^b^
200 ppm ABBE	1.81 ± 0.05 ^a^	9.31 ± 0.25 ^b^	8.41 ± 0.39 ^b^	8.26 ± 0.34 ^b^	7.72 ± 0.22 ^b^
500 ppm ABBE	1.81 ± 0.05 ^a^	8.39 ± 0.32 ^d^	7.73 ± 0.28 ^c^	7.62 ± 0.18 ^b^	7.49 ± 0.17 ^b^
800 ppm ABBE	1.81 ± 0.05 ^a^	6.62 ± 0.17 ^e^	6.47 ± 0.12 ^d^	5.91 ± 0.21 ^c^	5.78 ± 0.19 ^c^
200 ppm PBBE	1.81 ± 0.05 ^a^	9.47 ± 0.37 ^b^	8.69 ± 0.35 ^b^	8.41 ± 0.29 ^b^	7.94 ± 0.24 ^b^
500 ppm PBBE	1.81 ± 0.05 ^a^	8.54 ± 0.29 ^c^	8.46 ± 0.36 ^b^	8.02 ± 0.27 ^b^	7.63 ± 0.25 ^b^
800 ppm PBBE	1.81 ± 0.05 ^a^	6.78 ± 0.14 ^e^	6.65 ± 0.16 ^d^	6.42 ± 0.21 ^c^	5.97 ± 0.18 ^c^

C: Control sample. One-way ANOVA test was used to compare the means differences registered for each heating period among the oil samples supplemented with BHT and different doses of freeze-dried extracts of blueberry byproducts, relative to the control sample; data within the same column sharing different superscripts are significantly different (*p* < 0.05); data within the same column sharing the same superscripts are not significantly different (*p* > 0.05). Results are expressed as the average value of three independent analyses ±SD.

**Table 4:** The effect of sunflower oil supplementation with BHT and freeze-dried blueberry byproducts extracts on the *p*-Anisidine value (*p*-AV) during high-temperature heating.

Sample	*p*-AV				
	Heating Time (h)				
	0	3	6	9	12
C	2.49 ± 0.15 ^a^	47.86 ± 2.07 ^a^	56.98 ± 1.93 ^a^	62.27 ± 2.21 ^a^	69.84 ± 3.49 ^a^
200 ppm BHT	2.49 ± 0.15 ^a^	40.17 ± 2.28 ^b^	50.39 ± 2.43 ^a^	54.67 ± 2.17 ^a^	58.85 ± 2.52 ^b^
200 ppm ABBE	2.49 ± 0.15 ^a^	44.74 ± 2.86 ^a^	53.38 ± 3.09 ^a^	58.23 ± 3.14 ^a^	63.76 ± 3.32 ^a^
500 ppm ABBE	2.49 ± 0.15 ^a^	41.37 ± 2.38 ^a^	50.08 ± 2.23 ^a^	55.42 ± 3.18 ^a^	58.51 ± 2.92 ^b^
800 ppm ABBE	2.49 ± 0.15 ^a^	37.95 ± 1.87 ^d^	48.78 ± 2.42 ^b^	52.83 ± 2.65 ^b^	55.16 ± 1.96 ^c^
200 ppm PBBE	2.49 ± 0.15 ^a^	45.54 ± 2.36 ^a^	53.96 ± 2.48 ^a^	60.22 ± 3.54 ^a^	65.39 ± 4.02 ^a^
500 ppm PBBE	2.49 ± 0.15 ^a^	42.11 ± 2.93 ^a^	52.03 ± 2.82 ^a^	56.15 ± 3.35 ^a^	58.69 ± 3.44 ^b^
800 ppm PBBE	2.49 ± 0.15 ^a^	38.29 ± 2.16 ^c^	49.25 ± 3.01 ^b^	54.47 ± 2.75 ^a^	57.81 ± 3.26 ^b^

C: Control sample. One-way ANOVA test was used to compare the means differences registered for each heating period among the oil samples supplemented with BHT and different doses of freeze-dried extracts of blueberry byproducts, relative to the control sample; data within the same column sharing different superscripts are significantly different (*p* < 0.05); data within the same column sharing the same superscripts are not significantly different (*p* > 0.05). Results are expressed as the average value of three independent analyses ±SD.
